# Coenzyme Q10 Improves Functional and Structural Parameters of Dairy Goat Sperm During Cooling and Cryopreservation

**DOI:** 10.3390/antiox15060655

**Published:** 2026-05-22

**Authors:** Ranadheer Narlagiri, Abdallah M. Shahat, Courtney Henry, Ashvini Pawar, Niki C. Whitley, Iman B. Shaheed, Mahipal Singh, Brou Kouakou, Irina A. Polejaeva, Adel R. Moawad

**Affiliations:** 1 Animal Science Program, College of Agriculture, Family Sciences, and Technology, Fort Valley State University, Fort Valley, GA 31030, USA; rnarlagi@wildcat.fvsu.edu (R.N.); abdallah.mohamed@fvsu.edu (A.M.S.); chenry8@wildcat.fvsu.edu (C.H.); ashvini.pawar@fvsu.edu (A.P.); whitleyn@fvsu.edu (N.C.W.); singhm@fvsu.edu (M.S.); kouakoub@fvsu.edu (B.K.); 2 Department of Theriogenology, Faculty of Veterinary Medicine, Cairo University, Giza 12211, Egypt; 3 Department of Pathology, Faculty of Veterinary Medicine, Cairo University, Giza 12211, Egypt; imanshaheed@cu.edu.eg; 4 Department of Animal, Dairy and Veterinary Sciences, Utah State University, Logan, UT 84322, USA; irina.polejaeva@usu.edu

**Keywords:** goat bucks, cooling, cryopreservation, CoQ10, semen quality, mitochondria, sperm ultrastructure

## Abstract

Cryopreservation of gametes is crucial for conserving genetic diversity in livestock and endangered species, but the process can significantly impair sperm quality due to oxidative stress. Our aim was to evaluate the impacts of coenzyme Q10 (CoQ10) supplementation on the in vitro quality of cooled and cryopreserved goat semen. Semen samples collected from six mature Saanen bucks were pooled then diluted with AndroMed^®^ semen extender to a final concentration of 800 × 10^6^ sperm/mL. Diluted semen was supplemented with 0, 1, 2, 5, 10, and 20 µM CoQ10. Extended semen was either cooled at 4 °C for 72 h or cryopreserved using a Styrofoam box in which the straws were arranged on the freezing rack and placed 4 cm over the liquid nitrogen (LN_2_) for 10 min then stored in a LN_2_ tank for one-week before being thawed at 37 °C for 30 sec. Sperm quality, including total and progressive motility, sperm kinematics, live sperm %, and sperm membrane integrity, was assessed at 0 h (fresh semen), and 24, 48, and 72 h post-cooling. For post-thaw sperm, we evaluated the same parameters plus acrosome integrity, mitochondrial activity, lipid peroxidation, and sperm ultrastructural changes using scanning electron microscopy (SEM). The pooled semen sample was considered the experimental unit for all treatments. Cooled semen data were analyzed using a General Linear Model (GLM) with univariate analysis, followed by Tukey’s test for multiple comparisons. In contrast, data from frozen–thawed semen were analyzed using one-way analysis of variance (ANOVA) followed by Tukey’s test. CoQ10 supplementation at 10 and 20 µM significantly (*p* < 0.05) improved sperm motility, viability, and membrane integrity in cooled and frozen–thawed semen in comparison with the control group (0 µM CoQ10). Moreover, the same concentrations significantly (*p* < 0.05) enhanced acrosome integrity, mitochondrial activity, and reduced the percentages of sperm with lipid peroxidation in frozen–thawed semen. Furthermore, 10 and 20 µM CoQ10 significantly mitigated the ultrastructural defects in frozen–thawed spermatozoa. In conclusion, CoQ10 supplementation during the cooling and cryopreservation of dairy goat semen significantly improved sperm quality. Among the tested concentrations, 10 and 20 µM exhibited the most favorable outcomes.

## 1. Introduction

Artificial insemination (AI) has prospective applications in goat breeding systems. It enables the assessment of the genetic values of bucks across various herds and facilitates the dissemination of superior genetics while diminishing sanitary risks [1]. As the number of inseminated females increases, goat breeding programs advance rapidly, leading to improved fertility rates. Nevertheless, the application of AI remains limited in many dairy goat breeds, thereby constraining the genetic progress of these programs. In goats, AI is predominantly performed using semen chilled to 4 °C. The use of cooled semen is especially common during the non-breeding season; however, its optimal viability is generally restricted to the first 24 h of storage, making short-term functionality a major limitation of this approach [2,3].

While cryopreservation is a useful technique for storing sperm for an extended period, it is associated with a cold shock that disrupts the sperm plasma membrane, reducing sperm viability and fertility potential, and possibly lowering the effectiveness of AI [4]. During cryopreservation, excessive amounts of reactive oxygen species (ROS) are produced, this damages the methylene group of phospholipids in the sperm membrane and eventually causes lipid peroxidation [5]. Goat spermatozoa are highly susceptible to oxidative stress during cooling and freezing procedures mainly due to the high quantity of polyunsaturated fatty acids (PUFAs) in their plasma membranes [6,7].

To enhance reproductive success, researchers have tried to optimize cooling and cryopreservation semen extenders for small ruminants [8,9]. However, stressful conditions of cooling and freezing procedures produce structural and metabolic alterations in sperm which lower the quality and fertility performance after AI [10,11]. Optimization of these techniques, therefore, is required to enhance the quality of buck semen following cooling and cryopreservation processes.

Antioxidant compounds aid in mitigating the detrimental effects of excessive ROS generated during cryopreservation of mammalian sperm [12,13,14,15]. Although glutathione peroxidase (GPx), superoxide dismutase (SOD), and catalase (CAT) are endogenous antioxidants found in buck sperm [16], their quantities are insufficient to scavenge ROS generated during cooling, freezing and thawing processes [16,17]. Therefore, providing exogenous antioxidant molecules may preserve sperm viability during the chilling and cryopreservation procedures.

Coenzyme Q10 (CoQ10), ubiquinone, is a potent antioxidant localized in the inner mitochondrial membrane of the cell [18]. CoQ10 is a vitamin-like substance that is soluble in lipids; it plays an essential role in cell metabolism [19]. CoQ10 reduced form is also present in the testis at comparatively high concentrations [20,21], suggesting its significant impacts on spermatogenesis and sperm quality. The availability of Coenzyme Q10 (CoQ10) has been recognized as a crucial factor of sperm energy metabolism, thereby influencing sperm morphology and functional competence through its central role in cellular energy generation [22,23]. Previous studies showed that fortified semen extender with CoQ10 during cryopreservation improved sperm quality in horses [24], roosters [25], cattle, buffalo [26], and sheep [27]; however, little is known about its effect on cooled and cryopreserved dairy goat semen. Therefore, the goal of the current research was to assess the impact of CoQ10 supplementation on the in vitro quality of cooled and cryopreserved dairy goat spermatozoa.

## 2. Materials and Methods

### 2.1. Animals and Semen Collection

All animal use protocols were approved by the Fort Valley State University (FVSU, Fort Valley, GA, USA) Agricultural and Laboratory Animal Care and Use Committee (ALACUC, approval number SU-R-01-2024). Six mature Saanen bucks (2–3 years) were kept together in a barn at FVSU Dairy Goat Research Center. Bucks were collected from a defined local population under appropriate state and institutional approvals. Only healthy animals with no visible signs of disease or injury were included. Animals were not genetically modified, and their genotype was not manipulated or altered. No animals had been subjected to prior experimental procedures before inclusion in this study. The bucks were fed the commercial pelleted goat feed (18% crude protein; Mid-GA Medicated Goat [28]. Feed, Mid Georgia Farm Services LLC, Leesburg, GA, USA) which consisted of ground corn, soybean meal, dehydrated alfalfa meal, and grain by-products, supplemented with cane molasses, salt, ammonium chloride, and diatomaceous earth. Mineral sources included calcium, phosphorus, magnesium, potassium, and trace elements include Fe, Zn, Mn, Cu, I, Co, and Se. The diet was medicated with decoquinate (13.6 g/ton) for the prevention of coccidiosis. They were also given water ad libitum. Semen samples were collected from each buck once per week by electroejaculation over an eight-week period (October to December 2024). Collected samples were examined for total motility (TM), progressive motility (PM), and sperm concentration using a computer-assisted sperm analysis (CASA) system with AndroVision^®^ software (version 1.2.2, Minitube, Verona, WI, USA), following a previously described method [29]. Briefly, semen samples were diluted at a ratio of 1:100 in AndroMed semen extender (Minitube, Verona, WI, USA). A 5 µL aliquot of the diluted sample was then loaded into a prewarmed Leja^®^ counting chamber (Leja Products, Minitube, Verona, WI, USA) with a depth of 20 µm. The slides were examined under a 20× phase-contrast objective, and an average of five randomly selected fields per sample was captured. CASA settings were optimized for buck spermatozoa and maintained constant throughout all analyses as follows: frame rate of 60 Hz; 30 frames acquired; minimum contrast threshold of 35; and minimum cell size of 4 pixels. The static cell gate was set at 5.0 µm/s, and spermatozoa with a straightness (STR) ≥ 75% were classified as progressively motile. Semen samples exhibiting at least 80% total motility, 70% progressive motility, and a concentration of ≥2.0 × 10^9^ sperm/mL were pooled and processed for subsequent experiments.

### 2.2. Semen Dilution, CoQ10 Supplementation, Cooling and Cryopreservation

**Figure 1:**
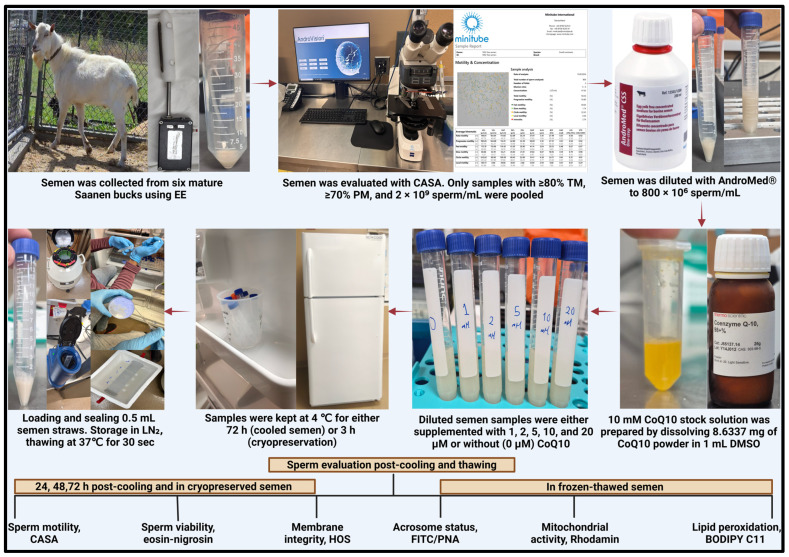
Schematic figure showing the experimental procedures. EE: electroejaculation; CASA: computer-assisted sperm analysis; TM: total motility; PM: progressive motility; CoQ10: coenzyme Q10; DMSO: dimethyl sulfoxide; LN_2:_ liquid nitrogen; HOS: hypo-osmotic swelling; FITC/PNA: fluorescein isothiocyanate/peanut agglutinin. Created in BioRender. Moawad, A. (2026) https://BioRender.com/50x6k87.

Pooled semen was diluted with AndroMed^®^ extender (Minitube, Verona, WI, USA) to a final sperm concentration of 800 × 10^6^ sperm/mL. The diluted semen was then divided into six aliquots corresponding to CoQ10 supplementation levels of 0, 1, 2, 5, 10, or 20 µM. A 10 mM CoQ10 stock solution was prepared by dissolving 8.6337 mg of CoQ10 powder (Thermo Fisher Scientific^®^, Waltham, MA USA; Cat. No. J65137.14) in 1 mL of dimethyl sulfoxide (DMSO). The stock solution was kept at −20 °C in Eppendorf tubes wrapped with aluminum foil to protect it from light. Diluted semen samples were fortified with CoQ10 at final concentrations of 1, 2, 5, 10, or 20 µM, or left unsupplemented (0 µM, control). To maintain a constant sperm concentration and achieve the desired CoQ10 concentrations, the appropriate volume of diluted semen was removed prior to the addition of the corresponding volume of CoQ10 solution. Samples were then gently but thoroughly mixed to ensure uniform distribution of CoQ10 throughout the semen. For cooling, diluted semen samples were kept in a water bath at 4 °C for 72 h. The quality of cooled semen, including sperm TM, PM, kinematics, viability, abnormalities, and membrane integrity, was assessed at 24, 48, and 72 h post-cooling. For cryopreservation, diluted semen samples were initially cooled at 4 °C for 3 h. Afterwards, the samples were loaded into 0.5 mL French semen straws and placed horizontally at a height of 4 cm above liquid nitrogen (LN_2_) in a Styrofoam box for 10 min to allow vapor freezing. The straws were then placed directly into LN_2_ and stowed for one week [30,31]. Then, frozen straws were thawed in a water bath at 37 °C for 30 s. Post-thaw spermatozoa were evaluated for the same parameters assessed in cooled semen, in addition to acrosome integrity, mitochondrial activity, lipid peroxidation and ultrastructure using scanning electron microscopy (SEM). A schematic diagram showing the experimental approach is presented in Figure 1.

### 2.3. Evaluation of Sperm Motility and Kinematics

Sperm TM, PM, and kinematic parameters—including curvilinear velocity (VCL), straight-line velocity (VSL), average path velocity (VAP), curvilinear distance (DCL), straight-line distance (DSL), amplitude of lateral head displacement (ALH), linearity (LIN), and straightness (STR)—in both cooled and frozen–thawed semen were evaluated using the CASA system and AndroVision^®^ software, as described in Section 2.1.

### 2.4. Evaluation of Sperm Viability and Abnormalities

**Figure 2:**
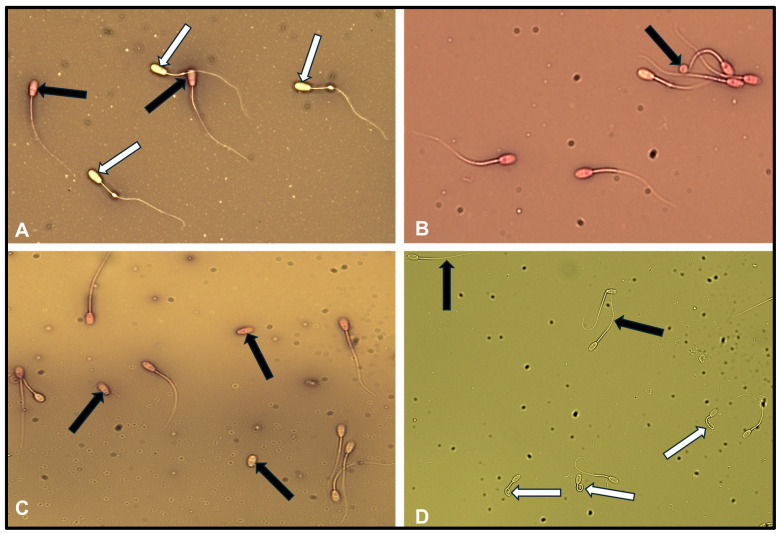
(**A**) Saanen goat spermatozoa stained with eosin–nigrosin showing live (white arrow) and dead (black arrow). (**B**,**C**) Saanen goat spermatozoa stained with eosin–nigrosin showing morphological abnormalities (black arrows) (**B**) coiled sperm tail and (**C**) detached sperm head. (**D**) Saanen goat spermatozoa incubated with hypo-osmotic swelling (HOS) solution showing coiled tail (HOS+; white arrows; intact membrane) and straight tail (HOS−; black arrows; non-intact membrane).

Sperm viability and morphological abnormalities were assessed using eosin–nigrosin staining, following a previously described protocol [29]. Briefly, 10 µL of diluted semen was blended with eosin and nigrosine stains (10 µL each) on a glass slide. Subsequently, this mixture was smeared onto a slide and permitted to dry in air. Afterwards, slides were assessed under a phase-contrast microscope at 40× magnification. Two hundred spermatozoa were examined per sample over various microscopic fields. Spermatozoa lack the stain uptake were recorded as viable (Figure 2A). Furthermore, the proportion of morphologically abnormal spermatozoa (Figure 2B,C) was recorded.

### 2.5. Evaluation of Sperm Membrane Integrity (Hypo-Osmotic Swelling (HOS) Test)

The functional integrity of sperm membrane was assessed by the HOS test according to the method described previously [32]. Briefly, 50 µL of extended semen was mixed with 1 mL of 125 mOsm/L HOS solution composed of 1.351 g fructose plus 0.753 g sodium citrate dihydrate in 100 mL of sterile deionized water. The mixture was then incubated for 45 min at 37 °C. After that, 15 µL of this mixture were carefully dropped onto a microscope slide and covered with a cover slip. The slides were examined under a phase contrast microscope with a 40× magnification. Two hundred spermatozoa were examined, and the percentage of sperm with coiled tails (HOS+, Figure 2D) was calculated as an indication of the intactness of the plasma membrane [29].

### 2.6. Evaluation of Sperm Ultrastructure by Scanning Electron Microscopy (SEM)

To examine the impacts of cryopreservation and CoQ10 supplementation on goat sperm ultrastructure, fresh (unfrozen), 0 (control), 10 and 20 µM CoQ10 frozen–thawed semen samples were washed three times with phosphate-buffered saline (PBS), with centrifugation at 4000 rpm for 5 min at each step. After centrifugation, the supernatant from each sample was discarded, and the resulting pellets were fixed in 2.5% glutaraldehyde in 0.1 M PBS (pH 7.4) for 8 h at 4 °C [33,34]. Following fixation, the samples were again centrifuged at 4000 rpm for 5 min and the supernatant was removed. Samples were then sequentially dehydrated through 30%, 50%, 70%, 90%, and 100% ethanol solutions for 10 min each. The dehydrated specimens were then transferred into microporous specimen capsules and subjected to critical point drying using a LEICA EM CPD 300 system (Leica Microsystems, Wetzlar, Germany) for 2 h. After drying, the capsules were carefully opened, and sections from the base were mounted onto carbon-coated grids [33,34]. The mounted samples were sputter-coated with gold for 2 min and subsequently evaluated using a scanning electron microscope (HITACHI S-3400N, Toyama, Japan). The SEM experiments were conducted at the Center for Ultrastructural Research (CURE), Fort Valley State University, Fort Valley, GA, USA.

### 2.7. Evaluation of Sperm Acrosome Status

**Figure 3:**
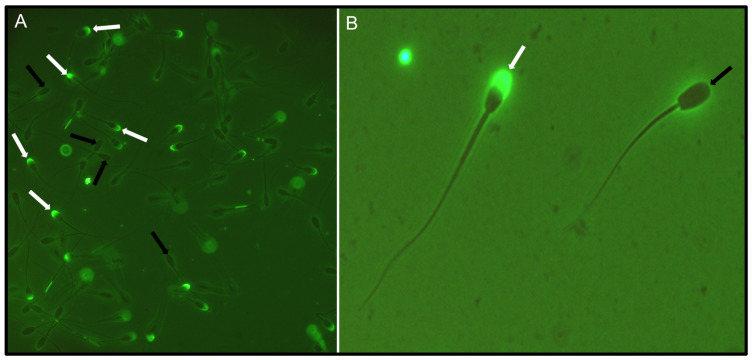
(**A**,**B**) Saanen goat spermatozoa stained with FITC-PNA fluorescence stain exhibiting intense and uniform green fluorescence on the acrosomal cap (sperm with intact acrosome, white arrows), while sperm that do not exhibit the same pattern were considered not-intact acrosome (black arrows).

The acrosome status was evaluated by using Hoechst 33342 (3.7%) and peanut agglutinin lectin labeling conjugated with fluorescein isothiocyanate (FITC-PNA, 6.6%) stain (Minitube, Verona, WI, USA) and the staining procedure was done as recommended by the company. Briefly, 27 µL of the ready to use stain was mixed carefully with 50 µL of frozen–thawed semen in a small Eppendorf tube and kept in the dark for 20 min at 38 °C. Consequently, a 10 µL drop from the mixture was added onto a microscope slide and covered with a coverslip. Slides were examined under a fluorescence microscope (Leica Microsystems, DM6 B, Wetzlar, Germany) using a DAPI/Hoechst (excitation: 375–435 nm; emission: 450–490), and GFP/FITC filters (excitation: 450–490 nm; emission: 500–550 nm) and a 40× objective. Two hundred spermatozoa were assessed and those exhibited intense and uniform green fluorescence (Figure 3) on the acrosomal cap were considered intact [35].

### 2.8. Evaluation of Mitochondrial Activity

Mitochondrial activity in the spermatozoa was evaluated by the Hoechst 33342 (2.5%)/Rhodamine 123 (0.5%) stain (Minitube, Verona, WI, USA) as recommended by the company. A total of 4 µL of the ready to use staining solution was mixed with 50 µL of the frozen–thawed semen sample in an Eppendorf tube and kept at 38 °C for 20 min in the dark. The samples were then centrifuged at 800 *g* for 4 min. Following centrifugation, the supernatant was removed, and the pellet was washed with 25 µL of phosphate-buffered saline (PBS) for 5 min at 38 °C. After that, 10 µL from the mixture were placed onto a microscope slide and covered with a coverslip. Slides were examined under a fluorescence microscope (Leica Microsystems DM6 B, Germany) using a DAPI/Hoechst (excitation: 375–435 nm; emission: 450–490), and GFP filter (excitation: 450–490 nm; emission: 500–550 nm) and a 40× objective. At least two hundred spermatozoa were evaluated and the proportion of spermatozoa showing green fluorescence in the midpiece (active mitochondria) was recorded.

### 2.9. Evaluation of Lipid Peroxidation

Lipid peroxidation levels in frozen–thawed sperm were determined by BODIPY 581/591 C11 probe (Thermo Fisher Scientific^®^, Waltham, MA, USA) according to company instructions. Briefly, 2 µL of BODIPY 581/591 C11 (2 µM final concentration) was mixed with 100 µL of frozen–thawed semen in a small Eppendorf tube and incubated at 38 °C for 30 min in the dark. The samples were then centrifuged at 800 *g* for 4 min. After removing the supernatant, the pellet was washed with 25 µL PBS. Then, 15 µL of the washed samples were placed on a glass slide, covered with a coverslip. Spermatozoa were examined under a fluorescence microscope (Leica Microsystems DM6 B, Germany) using a GFP filter (excitation: 450–490 nm; emission: 500–550 nm) and a 40× objective. Two hundred spermatozoa were assessed and the percentages of those that displayed intense green fluorescence in the midpiece were recorded as lipid peroxidation positive sperm.

### 2.10. Statistical Analysis

The pooled semen sample was considered the experimental unit for all treatments. The number of replicates for each treatment group was eight. Normality of data distributions was detected. Moreover, data variance was uniform (based on Levene’s tests). Data were presented as means ± standard error of the means (SEM). Cooled semen data were analyzed for the main effects (treatment and time) and their interactions (treatment×time) using General Linear Model (GLM) with univariate analysis, followed by Tukey’s post hoc test for multiple comparisons among treatment and time points. One-way analysis of variance (ANOVA), followed by Tukey’s test, was used for the analysis of frozen–thawed semen data. *p* < 0.05 was considered significant. The IBM SPSS 27.0 Software Package (IBM Corp., New York, NY, USA) was used to conduct statistical analysis.

## 3. Results

### 3.1. Effect of CoQ10 Supplementation on the Motility and Kinematic Parameters of Cooled Goat Spermatozoa

As shown in Table 1, irrespective to CoQ10 supplementation, sperm total and progressive motility significantly (*p* < 0.05) reduced in a time dependent manner with the lowest values seen at 72 h post-cooling. At 24 h and 48 h post-cooling, 2 µM CoQ10 improved (*p* < 0.05) TM as compared with the control (0 µM CoQ10) group. However, at 72 h post-cooling, sperm TM was higher (*p* < 0.05) in 10 and 20 µM CoQ10-supplemented groups than the control one (51.7% and 51.5% vs. 42.1%, respectively). Moreover, 10 and 20 µM CoQ10 supplementation significantly (*p* < 0.05) improved PM compared to the control group at all time points. There was a time effect (*p* < 0.05), however, there were no treatment effects (*p* > 0.05), furthermore, no treatment×time interaction was observed (*p* > 0.05) for both TM and PM (Table 1). Regarding sperm kinematics, all kinematic parameters decreased (*p* < 0.05) with cooling time and the lowest values were reported at 72 h post-cooling. At 24 h, CoQ10 supplementation at 2, 5, and 10 µM improved (*p* < 0.05) VCL values in comparison with the control (0 µM CoQ10). While at 48 h no significant effects of CoQ10 supplementation were observed on VCL. At 72 h, VCL values were the highest at 1 µM CoQ10 supplementation. For VSL, at 24 h post-cooling the values were significantly (*p* < 0.05) lower in CoQ10-supplemented groups than in the control group. However, at 48 h and 72 h post-cooling, no significant differences were observed between the control and CoQ10-supplemented groups. For VAP and DCL, values significantly (*p* < 0.05) decreased in CoQ10-supplemented groups as compared with the control at 24 h post-cooling. However, at 48 h, 1, 2, and 5 µM CoQ10 supplementation significantly (*p* < 0.05) improved VAP levels in comparison with the controls and other CoQ10 groups. At 72 h, the highest value of VAP was seen in the 1 µM CoQ10 group. For DSL, values significantly (*p* < 0.05) decreased in CoQ10-supplemented groups as compared with the controls at 24 h and 72 h post-cooling. However, at 48 h, the DSL level was higher (*p* < 0.05) in the 2 µM CoQ10 group than in the others. ALH and LIN values were significantly (*p* < 0.05) lower in CoQ10-supplemented groups than in the control at 24 h post-cooling. Meanwhile, no significant differences were observed between the control and CoQ10-treated groups at 48 h and 72 h. Finally, STR levels did not change significantly among the control and CoQ10 groups at all time points (Table 1). VAP, DCL, and DSL have treatment, time, and treatment×time interaction effects (*p* < 0.01).

### 3.2. Effect of CoQ10 Supplementation on Viability, Membrane Integrity and Abnormalities of Cooled Dairy Goat Spermatozoa

The percentages of live spermatozoa and sperm with intact plasma membranes (HOS+) significantly reduced (*p* < 0.05) in a time dependent manner with the lowest values observed at 72 h post-cooling. At 24 h and 48 h post-cooling, 2 µM and 5 µM CoQ10 supplementation significantly improved (*p* < 0.05) sperm viability and membrane integrity as compared with the control (0 µM) group. Meanwhile, at 72 h post-cooling, the highest live sperm % (59.8%) was observed in the 10 µM CoQ10-supplemented group, which was higher (*p* < 0.05) than the control (52.0%). The highest value of HOS+ sperm (58.5%) was seen in the 20 µM CoQ10-supplemented group (Table 2). Regarding sperm abnormalities, the percentages of abnormal sperm increased (*p* < 0.05) with cooling time and the highest values were reported at 72 h post-cooling (11.6% to 15.0% at 24 h vs. 27.1% to 39.0% at 72 h, respectively). At the same time, CoQ10 supplementation had no effects (*p* > 0.05) on sperm abnormalities (Table 2). Statistical analysis showed that no treatment effect and treatment×time interaction (*p* > 0.05), however there was time effect (*p* < 0.05) on sperm viability, membrane integrities and sperm abnormalities.

### 3.3. Effect of CoQ10 Supplementation on the Motility, Viability, Abnormalities, and Membrane Integrity of Frozen–Thawed Dairy Goat Spermatozoa

As shown in Table 3, supplementing semen extender with CoQ10 increased sperm total and progressive motility in a dose-dependent manner with the highest values reported at 20 µM (26.0% vs. 47.7%, for TM and 20.7% vs. 43.9% for PM in 0 and 20 µM CoQ10 groups, respectively (*p* < 0.05)). No significant differences were observed in sperm viability and total sperm abnormalities among the control and CoQ10-supplemented groups (percentages of live sperm ranged from 57.5% to 63.1% and abnormal sperm from 23.1% to 26.8%). The percentage of HOS+ sperm was higher (*p* < 0.05) for the 20 µM CoQ10-supplemented group (73.6%) than the 0 µM one (control, 59.2%).

### 3.4. Effect of CoQ10 Supplementation on Ultrastructure of Frozen–Thawed Dairy Goat Spermatozoa

**Figure 4:**
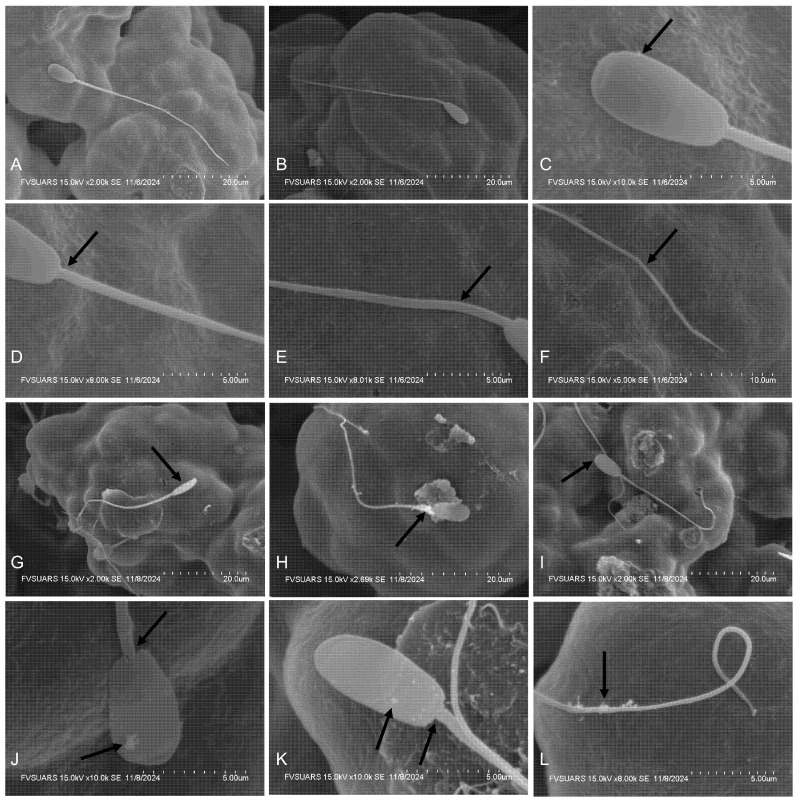
Scanning electron microscopy (SEM) for fresh (**A**–**F**) and frozen–thawed (**G**–**L**) Saanen goat spermatozoa. (**A**,**B**) Normal sperm with a homogenous plasma membrane. (**C**) Normal and oval sperm head with intact acrosome (black arrow). (**D**,**E**) Midpiece with intact plasma membrane and neck (black arrow). (**F**) Normal tail attaching areas (black arrow). (**G**) Defective sperm head (black arrow). (**H**) deformity in the midpiece (black arrow). (**I**) Ruptured acrosome (black arrow). (**J**,**K**) Wrinkled plasma membrane in the head region and ruptured membrane at the midpiece and membrane irregularities at the neck (black arrow). (**L**) Damaged tail area (black arrow).

Scanning electron microscopy (SEM) for fresh (unfrozen) spermatozoa revealed that spermatozoa had normal surface morphology with a homogenous plasma membrane (Figure 4A,B). The sperm head was normal and oval in shape with an intact acrosome (Figure 4C). The plasma membrane in the midpiece was intact. No deformities were noticed in the membrane near the neck (Figure 4D,E) and tail attaching regions (Figure 4F). In frozen–thawed spermatozoa, various defects include deformities in the sperm head (Figure 4G), midpiece (Figure 4H), and sperm acrosome (Figure 4I), were noticed. Moreover, some spermatozoa showed wrinkled membranes in the head region and integral regions of membrane rupture at the midpiece and membrane irregularities at the neck (Figure 4J,K). The tail area of the frozen–thawed sperm appeared damaged (Figure 4L). CoQ10 supplementation at 10 and 20 µM significantly mitigated these ultrastructural defects in frozen–thawed spermatozoa.

### 3.5. Effect of CoQ10 Supplementation on Acrosome Integrity, Mitochondrial Activity, and Lipid Peroxidation of Frozen–Thawed Dairy Goat Spermatozoa

As shown in Table 3, the percentages of spermatozoa with intact acrosome and active mitochondria were greater (*p* < 0.05) in the 20 µM CoQ10 fortified group than in the control (80.7% vs. 68.8% for acrosome and 80.9% vs. 65.4% for mitochondria, respectively). However, the proportion of sperm with positive lipid peroxidation was lower (*p* < 0.05) in the 20 µM CoQ10 group in comparison with the control (13.6% vs. 34.6%, respectively).

### 3.6. Effect of CoQ10 Supplementation on Kinematics of Frozen–Thawed Dairy Goat Spermatozoa

No significant differences were observed in sperm kinematics parameters including VCL, VSL, VAP, DCL, DSL, ALH, LIN, and STR between the control and CoQ10-supplemented groups or among different CoQ10 concentrations (Table 4).

## 4. Discussion

Goat spermatozoa are enriched in PUFAs rendering them highly vulnerable to oxidative stress–induced injury through cooling and cryopreservation procedures [36]. During these procedures, sperm quality is diminished, mainly because of the excessive production of ROS [36]. Generation of ROS in cooled and frozen semen alters sperm membrane integrity, reduces sperm motility, viability and subsequently diminishes sperm fertility [37]. Therefore, antioxidant supplementation during sperm cooling and cryopreservation is imperative to maintain sperm functions [37]. Herein we found that supplementation of CoQ10 during cooling and cryopreservation of dairy goat semen improved sperm motility, % of live sperm, and membrane intactness compared to the control non-supplemented samples. In addition, CoQ10-treated sperm displayed higher mitochondrial potential, acrosome intactness, and reduced lipid peroxidation levels after freezing and thawing. Compared with commonly used antioxidants such as vitamin E and selenium, CoQ10 may provide a dual function as both an antioxidant and a key element of the mitochondrial electron transport chain [38], thereby offering additional support for sperm energy metabolism during cooling and cryopreservation. However, further investigations, including fertility assessments and in vivo studies, are required to confirm its practical reproductive benefits.

Advancements in cooled storage procedures have revolutionized the quality of buck sperm and subsequently the success of AI in goat breeding. However, cold-induced oxidative stress and lipid peroxidation remain critical factors in deteriorating sperm viability [37]. Adjusting cryoprotectant constituents has thus developed as a pivotal strategy to safeguard against this damage [36]. Herein, we demonstrated that cold storage of dairy goat sperm meaningfully decreased total and progressive motility, viability and membrane integrity in a time-dependent manner with a drastic reduction seen at 72 h post-cooling. Like our findings, previous studies showed that goat sperm motility, velocities, viability, and mitochondrial membrane potential declined gradually while the duration of storage at 4 °C or 5 °C increased [39,40,41]. Furthermore, other studies reported an increase in apoptotic-like changes in chilled goat sperm as the duration of storage increased [40]. Also, the response to oxidative stress diminished in chilled goat sperm as the liquid storage period increased [2]. These perturbations in the sperm quality could explain the low fertilization potential of cooled goat spermatozoa stored for more than 24 h [2]. One of the key objections to chilled sperm is the limited shelf life [2,39]. One approach to expanding the longevity of cooled spermatozoa is a temporary reduction in cellular metabolism [2]. Hypothermia can reduce cellular metabolic activity by slowing enzymatic reactions [2,42,43]. In this context, it has been reported that goat sperm stored at 5 °C had greater motility compared to those stored at 17 °C [2,44,45]. In the present study, we chose to store the sperm at 5 °C for 72 h. It is well established that the negative impacts of cold shock during sperm cooling could be overcome by adding antioxidants [2]. In the present study, we found that supplementing extended semen with CoQ10 through cooling at 4 °C improved TM and PM across all time points (24 h, 48 h, and 72 h). Among the various concentrations used, 10 and 20 µM showed the highest PM. The beneficial impacts of CoQ10 on cooled goat sperm motility have been previously described [37]. The authors concluded that sperm total and progressive motility were markedly higher in the 5 µM CoQ10-supplemented group compared with 0 (control), 1, 2, and 10 µM supplemented groups [37]. The discrepancies between our results and Mohajer et al. (2024) [37] findings could be attributed to the differences in cooling times (24 h, 48 h, and 72 h herein versus 25 h and 50 h in Mohajer et al. (2024) [37] studies). Our findings suggest that high concentrations of CoQ10 (up to 20 µM) are required to maintain goat sperm motility during long cold storage (up to 72 h). In rooster cooled semen, it has been shown that adding 5 µM CoQ10 to semen extender improved the total and progressive motility at 24 h and 48 h, and that 10 µM CoQ10 showed a detrimental effect. These findings are different from ours as we found that 10 and 20 µM CoQ10 exhibited the best effect. These discrepancies might be explained by the differences in the structure of rooster and buck sperm and by the high PUFA contents in goats’ sperm membrane, which increases the levels of lipid peroxidation under ROS-induced oxidative conditions [46]. This could explain why high concentrations of CoQ10 are essential to exert their protective effect. In boar semen, it has been demonstrated that adding 1 mM CoQ10 to the semen extender improved sperm motility and viability after cooling at 17 °C [47]. Herein, we also showed that all kinematic parameters involving VCL, VSL, VAP, DCL, DSL, ALH, LIN, and STR, declined with cooling time, with the lowest values observed at 72 h. The positive effects of CoQ10 supplementation were seen only on VCL at 24 h post-cooling with the highest values demonstrated in 1 µM concentration. To date, no universally accepted reference values exist for sperm kinetic parameters in livestock [48]. Nonetheless, small ruminant VCL is higher than that of bulls or men [49]. Goat sperm experience massive oxidative injury during cooling as indicated by a decrease in kinematic values over the storage period [50]. The higher TM, PM, and VCL observed in CoQ10-treated cooled sperm speculates that inclusion of CoQ10 during cooling had the ability to overcome the oxidative damage imposed on goat semen.

Regarding sperm viability and sperm membrane integrity (HOS+ %), our study showed that although 10 µM CoQ10 had the best results, 20 µM CoQ10 was still significantly higher than the control group (0 µM). Similar findings were reported in cooled spermatozoa from bucks [51], boar [47], and roosters [52]. In these latter studies, sperm quality decreased with increasing cooling time, as recorded in our study where 72 h cooled samples had the lowest sperm viability and membrane intactness.

Polyunsaturated fatty acids in sperm membranes make them susceptible to lipid peroxidation resulting from the freezing and thawing processes, reducing viability. Antioxidants are thus utilized in cryopreservation solutions to avoid lipid peroxidation and enhance cryopreservation efficacy [53]. CoQ10’s characteristics make it a beneficial supplement that can be used in conjunction with goat sperm freezing extenders. It has been reported that testis [54], and male accessory glands [55] produce a high quantity of CoQ10, suggesting its useful impact on sperm quality. In our study, frozen–thawed goat spermatozoa showed better quality with CoQ10 supplementation. For post-thaw sperm total motility, live sperm, membrane integrity, and active mitochondria percentages, 10 and 20 µM CoQ10 showed the best results in comparison with the control (0 µM) and other CoQ10 concentrations. In parallel, post-thaw sperm progressive motility and the percentage of sperm with intact acrosome were the best at 10 and 20 µM CoQ10 than the control (0 µM) and other CoQ10 concentrations. On the other hand, 10 and 20 µM CoQ10 showed the lowest post-thaw sperm lipid peroxidation values. Our results agreed with Khazravi et al. (2024) [30], who used CoQ10 at 0, 0.1, 1, 10, and 100 µM as additives into the bucks’ semen diluent. They showed that 10 µM produced the best results across all the studied parameters we mentioned above. Also, they recorded that 100 µM had a damaging impact on the sperm quality after freezing and thawing. Yousefian et al. (2018) [31] reported that 1 µM CoQ10 improved goat bucks’ sperm motility, viability, acrosome integrity, and membrane intactness after cryopreservation and demonstrated better results than 1.5 µM, which was not detrimental to the sperm. On the other hand, supplementation of the ram [52] and rooster [25] semen extender with CoQ10 improved all the above-mentioned parameters in the post-thaw ram sperm at 2 µM concentrations, but 5 and 10 µM concentrations showed a harmful effect on the post-thaw sperm quality in the ram which was not the case for roosters. These differences might be related to the variations in sperm membrane structure among the different species, the use of chemicals from different sources, and seasonal effects as the goat bucks and rams are seasonal breeders.

Saeed et al. (2016) [26] reported that CoQ10 at 30 µM gave the best post-thaw motility, live %, sperm membrane, and acrosome integrity in post-thaw cattle and buffalo semen. Moreover, research by Carneiro et al. (2018) [24] examined the effect of the CoQ10 (0, 25, 50, 75, and 100 µM) supplementation on the quality of cryopreserved stallion semen and they reported that 75 µM had the best post-thaw sperm motility, membrane integrity, mitochondrial activity, and the lowest lipid peroxidation. In humans, Tas et al. (2023) [56] reported that adding 25 µM CoQ10 to the freezing media enhanced the post-thaw sperm motility, viability, membrane integrity, and total antioxidant capacity. Both our results and those of Tas et al. (2023) [47] support the conclusion that CoQ10 supplementation during cryopreservation improves sperm quality across livestock and human species. Regarding sperm abnormalities, the proportions of sperm with abnormal morphology were comparable among the CoQ10-treated groups. In vitro manipulation of sperm appears to have no significant effect on morphology, as sperm abnormalities primarily originate during spermatogenesis [57]. These results are in agreement with previous studies demonstrating that in vitro manipulation does not affect the morphology of rooster spermatozoa [25]. However, in contrast, a negative impact of CoQ10 on sperm morphology following the cryopreservation has been reported in goat sperm [31]. Scanning electron microscopy analysis revealed pronounced ultrastructural damage in frozen–thawed spermatozoa compared with fresh control samples (Figure 4). The observed abnormalities included irregular head morphology, tail deformities, compromised plasma membrane integrity, membrane rupture, and disruption of the acrosomal structure. Consistent with our findings, the detrimental effects of cryopreservation on sperm ultrastructure have been widely documented across multiple species, including cattle [58], sheep [58], goats [58], and mice [59]. Herein, adding CoQ10 at concentrations of 10 and 20 µM during cryopreservation significantly alleviated these ultrastructural impairments in goat sperm. These results highlight the protective role of CoQ10 during cryopreservation, preserving not only conventional in vitro sperm quality parameters but also maintaining the structural integrity of spermatozoa. Similarly, previous studies have demonstrated that antioxidant supplementation can mitigate oxidative stress-induced ultrastructural damage in goat sperm [60].

**Figure 5:**
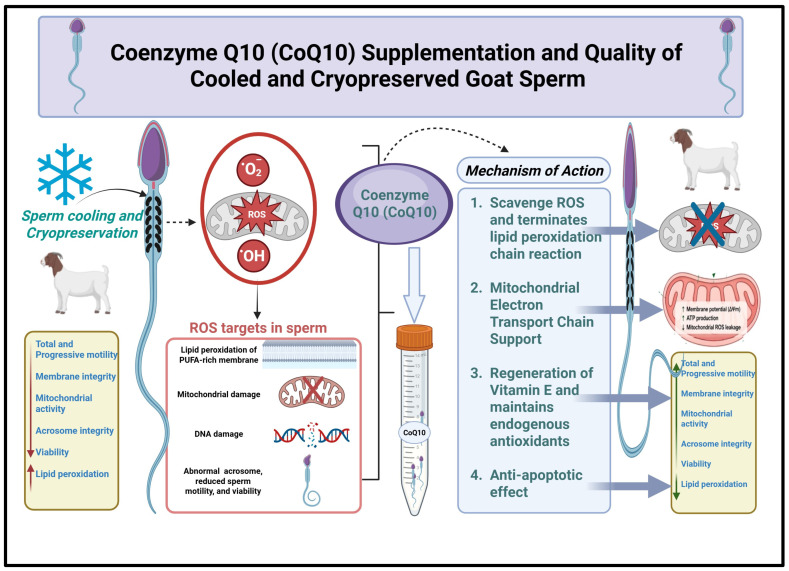
Schematic diagram showing the different mechanisms through which coenzyme Q10 (CoQ10) exerts its protective effect on cooled and cryopreserved goat spermatozoa. ROS: reactive oxygen species. PUFA: polyunsaturated fatty acid. ATP: adenosine triphosphate. Created in BioRender. https://BioRender.com/50x6k87.

Coenzyme Q10, also known as 1,4-benzoquinone, is an endogenous lipid-soluble molecule localized in the hydrophobic core of phospholipid bilayers across cellular membranes as well as in the mitochondria of most microorganisms and animals [18]. As mentioned above, CoQ10, a potent antioxidant, has demonstrated its ability to enhance sperm motility, live sperm %, and membrane intactness in the cryopreserved semen of various animal species. This component is essential for electron transport in numerous mitochondria. Since then, further cellular physiological benefits have been reported, such as antioxidant, signaling, and death prevention effects [18]. Turunen et al. (2004) [61] found that CoQ10’s lipophilic structure allows it to release phospholipids into the cell membrane, protecting the sperm membrane from changes. Moreover, membrane integrity is maintained by lowering the sperm’s vulnerability to peroxidation. Therefore, by preventing lipid peroxidation, CoQ10 preserves membrane functionality [62]. According to previous research [25,63], CoQ10 can also prevent the generation of hydroperoxides, safeguarding the sperm membrane from oxidative degradation [61]. Because CoQ10 is an energy carrier in the mitochondrion [62], its availability affects ATP synthesis and energy production within the cell [22]. CoQ10 can prevent mitochondrial membrane potential depolarization, ATP depletion, and caspase-9 activation. It also controls the mitochondrial permeability of the transition pores [61]. Therefore, it may make sense that the CoQ10 groups might exhibit fewer apoptotic-like alterations, given the better values of mitochondrial activity observed in the present study. A schematic diagram demonstrating the different mechanisms through which CoQ10 exerts its protective effect on cooled and frozen–thawed goat spermatozoa is presented in Figure 5. While this study provides clear evidence for the beneficial effects of CoQ10 supplementation on goat semen quality, several considerations should be acknowledged. The number of donor animals (n = 6), although consistent with similar experimental designs, may warrant expansion in future studies to further strengthen the generalizability of the findings. The study primarily assessed in vitro sperm quality parameters, which are well-established indicators of fertilizing potential; nonetheless, confirmation through in vitro fertilization (IVF) or in vivo fertility outcomes such as conception or kidding rates would further support the practical application of these findings. Finally, although key indicators of oxidative stress and mitochondrial function were examined, further characterization of antioxidant pathways and molecular mechanisms would provide additional insight into the mode of action of CoQ10.

## 5. Conclusions

CoQ10 supplementation during the cooling and cryopreservation of dairy goat semen significantly improved sperm quality. Among the tested concentrations, 10 and 20 µM exhibited the most favorable outcomes. These findings support a protective role of CoQ10 against oxidative damage, likely mediated through mitochondrial stabilization and antioxidant activity. Importantly, the improvement observed in vitro suggests enhanced fertilizing potential, which warrants further validation through IVF or AI trials.

## Figures and Tables

**Table 1:** Effects of CoQ10 supplementation on the motility and kinematic parameters of cooled dairy goat spermatozoa (means ± SEM).

Parmeter	CoQ10 (µM)	0 h	24 h	48 h	72 h
**TM** (**%**)	**0**	80.0 ± 1.2 ^A^	75.0 ± 1.2 ^bB^	62.4 ± 1.5 ^bC^	42.1 ± 1.8 ^bD^
	**1**		80.2 ± 2.3 ^aA^	77.0 ± 1.2 ^aA^	44.4 ± 1.2 ^bB^
	**2**		80.6 ± 2.0 ^aA^	79.2 ± 1.8 ^aA^	46.3 ± 2.0 ^bB^
	**5**		77.7 ± 1.3 ^bA^	78.8 ± 2.3 ^aA^	46.5 ± 1.5 ^bB^
	**10**		78.8 ± 1.5 ^bA^	75.7 ± 2.0 ^aA^	51.7 ± 1.3 ^aB^
	**20**		74.5 ± 2.0 ^bA^	75.2 ± 1.2 ^aA^	51.5 ± 2.0 ^aB^
**PM** (**%**)	**0**	68.0 ± 1.2 ^A^	65.8 ± 1.2 ^bA^	60.8 ± 1.2 ^cB^	42.0 ± 1.8 ^bC^
	**1**		69.5 ± 2.0 ^bA^	66.0 ± 1.5 ^bA^	38.7 ± 1.5 ^bB^
	**2**		76.0 ± 2.3 ^aA^	72.4 ± 1.8 ^aA^	41.0 ± 2.2 ^bB^
	**5**		69.6 ± 1.5 ^bA^	66.2 ± 2.3 ^bA^	41.2 ± 1.7 ^bB^
	**10**		76.0 ± 1.7 ^aA^	64.5 ± 2.0 ^bA^	47.1 ± 1.3 ^aB^
	**20**		77.2 ± 2.0 ^aA^	67.7 ± 1.8 ^bA^	49.8 ± 2.0 ^aB^
**VCL** (**μm/s**)	**0**	126.3 ± 1.2 ^A^	109.0 ± 1.2 ^cB^	97.4 ± 1.2 ^aC^	79.2 ± 1.2 ^bD^
	**1**		119.2 ± 2.0 ^bA^	101.8 ± 1.5 ^aB^	87.5 ± 1.5 ^aC^
	**2**		128.5 ± 2.3 ^aA^	97.6 ± 1.8 ^aB^	76.7 ± 1.8 ^bC^
	**5**		122.0 ± 1.5 ^aA^	102.6 ± 2.3 ^aB^	71.2 ± 2.2 ^cC^
	**10**		122.7 ± 1.7 ^aA^	85.5 ± 2.0 ^cB^	77.1 ± 1.3 ^bC^
	**20**		117.7 ± 2.0 ^bA^	92.7 ± 1.8 ^bB^	76.6 ± 2.0 ^bC^
**VSL** (**μm/s**)	**0**	128.1 ± 1.4 ^A^	84.6 ± 1.2 ^aB^	33.6 ± 1.2 ^aC^	31.8 ± 1.8 ^aC^
	**1**		44.2 ± 2.0 ^bA^	35.4 ± 1.5 ^aB^	32.0 ± 1.5 ^aB^
	**2**		47.7 ± 2.3 ^bA^	37.4 ± 1.8 ^aB^	27.0 ± 2.2 ^aC^
	**5**		45.6 ± 1.5 ^bA^	35.6 ± 2.3 ^aB^	27.2 ± 1.7 ^aC^
	**10**		45.7 ± 1.7 ^bA^	31.0 ± 2.0 ^aB^	29.1 ± 1.3 ^aB^
	**20**		46.2 ± 2.0 ^bA^	34.7 ± 1.8 ^aB^	28.5 ± 2.0 ^aB^
**VAP** (**μm/s**)	**0**	120.6 ± 1.2 ^A^	98.3 ± 1.2 ^aB^	43.8 ± 1.2 ^bC^	38.0 ± 1.8 ^aD^
	**1**		53.0 ± 2.0 ^cB^	45.6 ± 1.5 ^aC^	39.5 ± 1.5 ^aD^
	**2**		57.7 ± 2.3 ^bB^	46.2 ± 1.8 ^aC^	34.0 ± 2.2 ^bD^
	**5**		55.3 ± 1.5 ^bB^	46.0 ± 2.3 ^aC^	33.2 ± 1.7 ^cD^
	**10**		53.2 ± 1.7 ^cB^	39.2 ± 2.0 ^cC^	35.5 ± 1.3 ^bD^
	**20**		54.2 ± 2.0 ^cB^	43.0 ± 1.8 ^bC^	35.3 ± 2.0 ^bD^
**DCL** (**µm**)	**0**	60.8 ± 1.3 ^A^	53.2 ± 1.2 ^aA^	32.8 ± 1.2 ^bB^	28.0 ± 1.8 ^bC^
	**1**		40.0 ± 2.0 ^bA^	33.6 ± 1.5 ^aB^	29.5 ± 1.5 ^aC^
	**2**		42.0 ± 2.3 ^aA^	33.4 ± 1.8 ^aB^	26.2 ± 2.2 ^bC^
	**5**		40.0 ± 1.5 ^bA^	33.6 ± 2.3 ^aB^	24.2 ± 1.7 ^cC^
	**10**		41.2 ± 1.7 ^bA^	30.0 ± 2.0 ^bB^	26.8 ± 1.3 ^bC^
	**20**		41.5 ± 2.0 ^bA^	31.5 ± 1.8 ^bB^	26.1 ± 2.0 ^bC^
**DSL** (**µm**)	**0**	46.1 ± 1.3 ^A^	33.6 ± 1.2 ^aA^	11.2 ± 1.2 ^bB^	11.3 ± 1.8 ^aC^
	**1**		14.5 ± 2.0 ^cA^	11.4 ± 1.5 ^bB^	10.8 ± 1.5 ^bC^
	**2**		15.2 ± 2.3 ^bA^	12.4 ± 1.8 ^aB^	9.0 ± 2.2 ^bC^
	**5**		15.0 ± 1.5 ^bA^	11.6 ± 2.3 ^bB^	9.2 ± 1.7 ^bC^
	**10**		15.7 ± 1.7 ^bA^	10.7 ± 2.0 ^bB^	9.9 ± 1.3 ^bC^
	**20**		15.7 ± 2.0 ^bA^	11.6 ± 1.8 ^bB^	9.6 ± 2.0 ^bC^
**ALH** (**µm**)	**0**	2.6 ± 1.2 ^A^	2.5 ± 1.2 ^aA^	2.4 ± 1.2 ^aAB^	2.0 ± 1.8 ^aC^
	**1**		2.9 ± 2.0 ^bA^	2.5 ± 1.5 ^aB^	2.2 ± 1.5 ^aC^
	**2**		3.1 ± 2.3 ^bA^	2.3 ± 1.8 ^aB^	2.1 ± 2.2 ^aB^
	**5**		3.0 ± 1.5 ^bA^	2.5 ± 2.3 ^aB^	1.8 ± 1.7 ^aC^
	**10**		2.9 ± 1.7 ^bA^	2.1 ± 2.0 ^aB^	2.0 ± 1.3 ^aB^
	**20**		2.8 ± 2.0 ^bA^	2.2 ± 1.8 ^aB^	2.1 ± 2.0 ^aB^
**LIN** (**%**)	**0**	0.6 ± 0.3 ^A^	0.5 ± 0.1 ^aA^	0.3 ± 0.1 ^bB^	0.3 ± 0.1 ^bA^
	**1**		0.3 ± 0.1 ^bA^	0.3 ± 0.2 ^bA^	0.3 ± 0.5 ^bA^
	**2**		0.3 ± 0.2 ^bA^	0.3 ± 0.3 ^bA^	0.3 ± 0.2 ^bA^
	**5**		0.3 ± 0.3 ^bA^	0.3 ± 0.2 ^bA^	0.3 ± 0.2 ^bA^
	**10**		0.3 ± 0.3 ^bA^	0.3 ± 0.3 ^bA^	0.3 ± 0.2 ^bA^
	**20**		0.3 ± 0.2 ^bA^	0.3 ± 0.2 ^bA^	0.3 ± 0.3 ^bA^
**STR** (**%**)	**0**	0.9 ± 0.2 ^A^	0.8 ± 0.1 ^aAB^	0.7 ± 0.3 ^aB^	0.5 ± 0.2 ^aC^
	**1**		0.8 ± 0.2 ^aA^	0.7 ± 0.3 ^aA^	0.7 ± 0.2 ^aA^
	**2**		0.8 ± 0.1 ^aA^	0.7 ± 0.2 ^aA^	0.7 ± 0.2 ^aA^
	**5**		0.7 ± 0.3 ^aA^	0.7 ± 0.2 ^aA^	0.7 ± 0.2 ^aA^
	**10**		0.8 ± 0.2 ^aA^	0.7 ± 0.2 ^aA^	0.7 ± 0.3 ^aA^
	**20**		0.8 ± 0.1 ^aA^	0.7 ± 0.3 ^aA^	0.7 ± 0.1 ^aA^

^a–c^ denotes significant differences (*p* < 0.05) at different CoQ10 concentrations within the same column. ^A–D^ denotes significant differences (*p* < 0.05) between time points within the same row for the same CoQ10 concentration. 0 h represents semen samples before cooling (fresh samples). TM: total motility. PM: progressive motility. VCL (curvilinear velocity), VSL (straight line velocity), VAP (average path velocity), DCL (distance curved line), DSL (distance straight line), ALH (amplitude of lateral head displacement), LIN (linearity), STR (straightness). n = 8 for all groups.

**Table 2:** Effects of CoQ10 supplementation on viability, membrane integrity and total abnormalities of cooled dairy goat spermatozoa (means ± SEM).

Parameters	CoQ10	0 h	24 h	48 h	72 h
**Live sperm** (**%**)	**0**	82.5 ± 1.2 ^A^	77.6 ± 1.2 ^cB^	74.0 ± 1.2 ^bB^	52.0 ± 1.3 ^bC^
	**1**		81.2 ± 2.0 ^bA^	77.2 ± 1.5 ^bB^	51.0 ± 1.5 ^bC^
	**2**		86.0 ± 2.3 ^aA^	84.4 ± 1.3 ^aA^	43.0 ± 2.2 ^cB^
	**5**		83.6 ± 1.6 ^abA^	83.6 ± 2.3 ^aA^	43.5 ± 1.5 ^cB^
	**10**		81.2 ± 1.2 ^bA^	77.7 ± 2.0 ^bB^	59.8 ± 1.3 ^aC^
	**20**		82.7 ± 2.0 ^bA^	79.2 ± 1.5 ^aB^	53.3 ± 2.0 ^aC^
**Membrane integrity** (**HOS+ %**)	**0**	83.5 ± 1.2 ^A^	74.5 ± 1.2 ^cB^	70.4 ± 1.2 ^cB^	53.8 ± 1.5 ^aC^
	**1**		80.2 ± 2.0 ^bA^	78.4 ± 1.3 ^bB^	46.2 ± 1.3 ^bC^
	**2**		86.0 ± 2.2 ^aA^	80.0 ± 1.7 ^aB^	55.5 ± 2.1 ^aC^
	**5**		86.6 ± 1.5 ^aA^	81.2 ± 2.3 ^aA^	40.5 ± 1.5 ^cB^
	**10**		80.0 ± 1.3 ^bA^	76.0 ± 2.0 ^bB^	52.1 ± 1.3 ^aC^
	**20**		80.7 ± 1.8 ^bA^	76.5 ± 1.6 ^bB^	58.5 ± 1.8 ^aC^
**Total abnormalities** (**%**)	**0**	15.6 ± 1.2 ^A^	15.0 ± 1.2 ^bA^	17.0 ± 1.1 ^bA^	30.6 ± 1.8 ^aB^
	**1**		13.7 ± 2.0 ^aA^	14.6 ± 1.5 ^aA^	33.0 ± 1.3 ^bB^
	**2**		12.2 ± 2.2 ^aA^	14.2 ± 1.7 ^aA^	39.0 ± 2.1 ^bB^
	**5**		11.6 ± 1.4 ^aA^	14.6 ± 2.3 ^aA^	37.0 ± 1.7 ^bB^
	**10**		13.0 ± 1.5 ^aA^	17.7 ± 2.0 ^bA^	28.5 ± 1.2 ^aB^
	**20**		13.0 ± 2.0 ^aA^	16.0 ± 1.5 ^bA^	27.1 ± 2.0 ^aB^

^a–c^ denotes significant differences (*p* < 0.05) at different CoQ10 concentrations within the same column. ^A–C^ denotes significant differences (*p* < 0.05) between time points for the same CoQ10 concentration within the same row. 0 h represents semen samples before cooling (fresh samples). HOS: hypo-osmotic swelling. n = 8 for all groups.

**Table 3:** Effects of CoQ10 supplementation on the quality of frozen/thawed dairy goat spermatozoa (means ± SEM).

CoQ10 (µM)						
Parameter	0	1	2	5	10	20
**TM** (**%**)	26.0 ± 1.7 ^a^	30.3 ± 1.3 ^a^	36.2 ± 1.4 ^b^	42.4 ± 1.4 ^bc^	44.1 ± 1.5 ^c^	47.7 ± 1.3 ^c^
**PM** (**%**)	20.7 ± 1.3 ^a^	24.3 ± 1.0 ^b^	30.5 ± 1.2 ^c^	37.6 ± 1.2 ^d^	39.3 ± 1.1 ^de^	43.9 ± 1.1 ^e^
**Live sperm** (**%**)	57.5 ± 3.3 ^a^	59.4 ± 3.1 ^a^	60.6 ± 3.1 ^a^	62.5 ± 3.2 ^a^	63.6 ± 3.3 ^a^	63.1 ± 3.1 ^a^
**Total abnormalities** (**%**)	26.8 ± 1.8 ^a^	25.5 ± 2.0 ^a^	23.1 ± 1.8 ^a^	23.6 ± 1.9 ^a^	24.0 ± 1.8 ^a^	24.6 ± 1.9 ^a^
**HOS +** (**%**)	59.2 ± 2.6 ^a^	62.6 ± 2.7 ^ab^	69.4 ± 2.6 ^ab^	69.5 ± 2.2 ^b^	69.3 ± 2.0 ^b^	73.6 ± 1.9 ^b^
**Intact acrosome** (**%**)	68.8 ± 0.8 ^a^	72.6 ± 0.7 ^b^	75.0 ± 0.6 ^bc^	77.3 ± 0.7 ^c^	78.6 ± 0.7 ^cd^	80.7 ± 0.6 ^d^
**Active mitochondria** (**%**)	65.4 ± 0.7 ^a^	66.2 ± 0.8 ^a^	70.0 ± 0.8 ^b^	75.7 ± 0.9 ^c^	78.6 ± 0.7 ^cd^	80.9 ± 0.6 ^d^
**Lipid peroxidation +** (**%**)	34.6 ± 0.7 ^a^	25.3 ± 0.7 ^b^	21.3 ± 0.8 ^c^	20.6 ± 0.7 ^c^	16.3 ± 0.9 ^d^	13.6 ± 0.5 ^d^

^a–e^ denote significant differences (*p* < 0.05) among different concentrations of CoQ10 within the same row. TM: total motility. PM: progressive motility. HOS: hypo-osmotic swelling. n = 8 for all groups.

**Table 4:** Effects of CoQ10 supplementation on kinematics parameters of frozen/thawed dairy goat spermatozoa (means ± SEM).

CoQ10 (µM)						
Parameter	0	1	2	5	10	20
**VCL** (**μm/s**)	94.1 ± 1.2	93.9 ± 1.5	92.5 ± 1.8	79.8 ± 1.7	88.6 ± 1.7	87.6 ± 1.2
**VSL** (**μm/s**)	37.5 ± 1.2	39.2 ± 1.4	36.9 ± 1.5	32.6 ± 1.3	39.1 ± 1.2	37.6 ± 1.2
**VAP** (**μm/s**)	43.7 ± 1.2	45.3 ± 1.4	43.6 ± 1.5	38.8 ± 1.2	44.8 ± 1.6	43.3 ± 1.8
**DCL** (**μm**)	30.7 ± 1.2	30.9 ± 1.5	30.5 ± 1.4	27.6 ± 1.7	30.6 ± 1.6	29.9 ± 1.3
**DSL** (**μm**)	12.1 ± 1.4	12.6 ± 1.4	11.9 ± 1.4	11.1 ± 1.2	13.5 ± 1.4	12.9 ± 1.6
**ALH** (**μm**)	2.3 ± 0.1	2.3 ± 0.1	2.3 ± 0.1	2.0 ± 0.1	2.1 ± 0.1	2.1 ± 0.2
**LIN** (**%**)	0.43 ± 0.02	0.45 ± 0.02	0.43 ± 0.02	0.45 ± 0.02	0.48 ± 0.02	0.47 ± 0.03
**STR** (**%**)	0.85 ± 0.01	0.86 ± 0.01	0.84 ± 0.01	0.83 ± 0.01	0.86 ± 0.01	0.86 ± 0.01

VCL: curvilinear velocity. VSL: straight line velocity. VAP: average path velocity. DCL: distance curved line. DSL: distance straight line, ALH: amplitude of lateral head displacement, LIN: linearity. STR: straightness. n = 8 for all groups.

## Data Availability

The original contributions presented in this study are included in the article. Further inquiries can be directed to the corresponding author.

## Abbreviations

The following abbreviations are used in this manuscript:

TM	total motility
PM	progressive motility
GPx	glutathione peroxidase
SOD	superoxide dismutase
CAT	catalase
VCL	curvilinear velocity
VSL	straight line velocity
VAP	average path velocity
DCL	distance curved line
DSL	distance straight line
ALH	amplitude of lateral head displacement
LIN	linearity
STR	straightness
HOS	hypo-osmotic swelling
